# Commentary: Vitamin D status and tic disorder: a systematic review and meta-analysis of observational studies

**DOI:** 10.3389/fped.2024.1385212

**Published:** 2024-06-20

**Authors:** David E. Most

**Affiliations:** ^1^ School of Education, Colorado State University, Fort Collins, CO, United States; ^2^ Department of Biostatistics and Informatics, Colorado School of Public Health, Fort Collins, CO, United States

**Keywords:** vitamin D, tic disorders, scientific inference and reasoning, statistical inference abuse, meta-analytic thinking

A Commentary on Vitamin D status and tic disorder: a systematic review and meta-analysis of observational studies By Most DE (2024). Front. Pediatr. 12.1173741. doi: 10.3389/fped.2023.1173741

## Introduction

The goal of this commentary is to provide a constructive critique of some of the key findings of this interesting study regarding the clinical relationship between tic disorders (TDs) and vitamin D levels in children (1). Tic disorders were divided into the following three subgroups: transient tic disorder (TTD), chronic motor or vocal tic disorder (CTD), and Tourette syndrome (TS). The relationship of interest was evaluated by performing a systematic review and meta-analysis that combined information from 13 observational studies. The meta-analysis resulted in estimates of mean differences in serum vitamin D levels among all three TD subgroups, in addition to an overall comparison of children with TD and healthy controls (HCs). In the conclusion section of the abstract, after noting that the vitamin D level of children with TD was lower than that of healthy children, with respect to the comparisons among subtypes of TD, the authors state that “there was no difference between the subgroup.” The central concern of this commentary is that, for all the TD subgroup comparisons, the evidence does not seem to support such a conclusion.

## Evidence and meaning

What does the evidence seem to suggest? The key comparisons of interest are presented both in the results section of the abstract and in Section 3.3 of the article, which summarizes the meta-analysis of mean serum vitamin D concentrations. For the comparison of HC with TD, the point estimate is 6.64, which is consistent with the authors’ prose description that the vitamin D level of healthy children was higher than that of children with TD. However, for the comparisons between TTD and CTD, CTD and TS, and TTD and TS, the point estimates of differences in serum vitamin D levels are 3.84, 1.06, and 5.24, respectively, all of which are inconsistent with the claim that there were no TD subgroup differences. Even before considering uncertainty, it is arguable that the point estimates of the TD subgroup differences are not much smaller than the overall difference between HC and TD. For example, the estimated magnitude of two of the TD subgroup differences is more than half of the overall difference between HC and TD. None of the estimated TD subgroup differences are zero.

Why is there an inconsistency between the evidence and the interpretation of the results? The reason for the inconsistency is a common error in interpretation. The problem arises when one derives a substantive interpretation solely from a binary statistical declaration. A statistical claim of “no statistically significant difference” morphs into a scientific conclusion regarding the absence of evidence for a difference or simply of “no difference.” The authors’ interpretation of their results is based on such statistical declarations rather than clinical judgments regarding the magnitude of the estimated quantities of interest. This leads to the distinction between the existence of an overall difference in serum vitamin D levels between HC and TD and the absence of a difference between the TD subgroups. This distinction is problematic, as it is an error to argue that the difference between “significant” and “not significant” is clinically noteworthy (2). One puzzling discrepancy is that the authors note in the results section of the abstract that there was a statistically significant difference between the TTD and TS subgroups. However, the overarching clinical conclusion offered is one of no TD subgroup differences in serum vitamin D levels despite the aforementioned point estimates. In short, “we should never conclude there is “no difference” or “no association” just because a *p*-value is larger than a threshold such as 0.05 or, equivalently, because a confidence interval includes zero” (3).

What about uncertainty in the estimates of the differences between TD subgroups? The inclusion of 95% CIs in the abstract and Section 3.3 is appreciated and helpful for getting a handle on the magnitude of uncertainty in TD subgroup differences, although the authors do not seem to reference the CIs when making meaning of the evidence in their data. A consideration of the CIs suggests that the plausible true values for subgroup differences range from approximately 0 to 8 for TTD and CTD, 0 to 2 for CTD and TS, and 0 to 10 for TTD and TS. Given these interval estimates, while it is plausible that the true difference between the TD subgroups is zero, the ranges of plausible true differences between TD subgroups that are compatible with the data include a variety of values, many of which might be considered clinically significant. Many of the plausible true subgroup differences are larger than the point estimate of the overall difference in serum vitamin D levels between TD and HC. Acknowledging the meaning of this uncertainty supports the perspective that the evidence is inconsistent with a scientific or clinical interpretation that no difference was found between TD subgroups.

## Discussion

Confusing statistical inference with scientific inference is a century-old problem, and the focus of this commentary is a particular empirical example (4). Associated interpretational errors are ubiquitous in a variety of disciplines (3). The interpretation that there were no TD subgroup differences in serum vitamin D levels depends on mistakenly conflating the notion of a declaration regarding statistical significance (based, for example, on *p*-values of 0.09 and 0.06), with a clinical judgment about the nature of a difference. Instead of focusing on a binary declaration regarding whether the true differences between the TD subgroups could be zero, a better way to make meaning of these data might be to evaluate the magnitude of the estimated differences in serum vitamin D concentrations. Such an evaluation should be based on clinical expertise while simultaneously embracing statistical and scientific uncertainty. While methods of statistical inference can help quantify some types of uncertainty, accurate characterizations of cumulative knowledge, which are the primary purpose of systematic reviews and meta-analyses, depend on scientific summaries that have fidelity to the evidence.

## References

[1] Xiaoxia L Jilong J Xianrui C Yanhui C. Vitamin D status and tic disorder: a systematic review and meta-analysis of observational studies. Front Pediatr. (2023) 11:1173741. 10.3389/fped.2023.1173741 37325365 PMC10267821

[2] Gelman A Stern H. The difference between “significant” and “not significant” is not itself statistically significant. Am Stat. (2006) 60(4):328–31. 10.1198/000313006X152649

[3] Valentin A Sander G Blake M. Retire statistical significance. Nature (London). (2019) 567(7748):305–7. 10.1038/d41586-019-00857-9 30894741

[4] Wasserstein, Schirm AL Lazar NA. Moving to a world beyond “*p* < 0.05.”. Am Stat. (2019) 73(sup1):1–19. 10.1080/00031305.2019.1583913

